# Nature as a Source of Inspiration for the Structure of the Sydney Opera House

**DOI:** 10.3390/biomimetics7010024

**Published:** 2022-02-02

**Authors:** Juan Rey-Rey

**Affiliations:** Higher Technical School of Architecture, University of A Coruña, 15008 A Coruña, Spain; j.rey.rey@udc.es

**Keywords:** Sydney Opera House, biomimetics, shell structures, reinforced concrete shells, funicularity

## Abstract

Architects throughout the ages have looked to nature for answers to complex questions about the most appropriate structural forms for their buildings. This is the case of Jørn Utzon and the design of roof shells of the Sydney Opera House, in which the search for natural references was constant, from the nautical references in the initial design phases to the final spherical solution based on the analogy with an orange. This paper analyzes the influence of nature as a source of inspiration in this World Heritage building, assessing through FEM calculation models the suitability of the different solutions proposed and weighing up the influence of certain factors such as scale in this type of process. Through the calculation models developed, it has been possible to verify the poor performance of the initial designs compared to the power of the final solution, which, after more than 5 years of research by the design team headed by Utzon, was able to solve the enormous problem with a “simple” typological and geometric change.

## 1. Introduction

**Figure 1:**
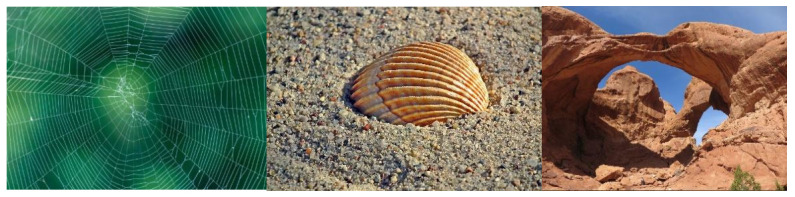
**Left**: spider web (photo by www.patternpictures.com; last accessed 2 December 2021); **center**: mollusk shell (photo by www.pixabay.com; last accessed 2 December 2021); **right**: Double O Arch in Utah’s Arches National Park: 22 m long sandstone arches (photo by Ken Lund).

Nature has always been a great source of inspiration for architectural and structural design, providing effective models through constant evolution and optimization for over 3.8 billion years. This mimicry of effective natural models has been particularly relevant in the case of structural systems. There are several examples of natural structures that have served as a source of inspiration for many constructions over time (Figure 1): eggshells, spider webs, various animal shells, bones, tree structures, natural antifunicular geological formations, etc.

As mentioned, it is a common strategy among architects and engineers to take inspiration from nature for the design of buildings and other constructions. Countless analogies can be found in the architecture of all ages. However, the formal creation of the field of knowledge in biomimetics is very recent. Specifically, the term “biomimetics” was proposed in the 1950s by the biophysicist and engineer Otto H. Schmitt to designate a new field of knowledge within biomedical engineering. In 1960, the term “bionics” (as a combination of the terms “biology” and “technics”) was also invented in the USA by Jack E. Steel, at a conference entitled “Bionics Symposium: Living Prototypes, the Key to New Technology” [1]. The term referred to the study of the functions and structures of biological systems as reference models for design in the field of engineering. The term “biomimicry” was proposed in the 1980s by the biologist Janine Benyus, author of the book *Innovation Inspired by Nature* [2]. Biomimicry is defined in her book as a new science that studies nature in order to imitate it or to draw inspiration from it to solve human problems [3].

Some well-known examples of biomimicry in architecture are Norman Foster’s Gherkin Tower (2004, London, UK) or The Eastgate Centre, designed by architect Mick Pearce (1966, Harare, Zimbabwe).

## 2. Methods

The working method chosen for the development of this research is the case study, taking the Sydney Opera House as an example on which to reflect on the potential of inspiration from natural forms in the field of architectural and structural design: its advantages as well as its risks.

The reasons for choosing the Sydney Opera House as a case study are various: it is an extraordinary example, a building ahead of its time in which scientific and technological advances such as computers were used in a pioneering way, but at the same time, it is a project with strong links to the past, to ancient civilizations, and, above all, to nature.

Through the vast bibliography available, the role of nature as the main source of inspiration for the structure of the roofs, iconic elements of the building, has been analyzed. Specific 3D calculation models have also been developed to evaluate the structural performance of the various proposals for the roof structures.

## 3. Biomimetics in Building Structures

### 3.1. Introduction

Architects and engineers throughout the ages have looked to nature for answers to complex questions about the most appropriate structural forms for their buildings. This translation of ideas from the natural source of inspiration to the concrete structural problem is not simple and has not always been successful.

It is possible to list several strategies for the application of biomimicry in the design of buildings and in their load-bearing structure in particular: the examination and application of nature’s materials, the symbolic or structural transfer of natural form, or the interrelation of the building structure with the environment, among others [1].

In the Sydney Opera House, we can find a number of examples of application of mainly the second strategy: symbolic or structural transfer of natural form, as explained in detail in Section 4.

### 3.2. Engineering and Nature

There is a clear analogy between nature and structural engineering, based on the search in both fields for minimum energy consumption to evolve towards the most efficient systems possible. The reflections of Beukers et al. are very interesting in this respect:

*“There is a duality between engineering and nature, which is based on minimum use of energy. This is because animals and plants, in order to survive in competition with each other, have evolved ways of living and reproducing using the least amount of resource. This involves efficiency both in metabolism and optimal apportionment of energy between the various functions of life. A similar situation obtains with engineering, where cost is usually the most significant parameter. It seems likely, then, that ideas from nature, suitably interpreted and implemented, could improve the energy efficiency of our engineering at many levels. This transfer of technology, variously called bionics, biomimetics or biognosis, should not be seen so much as a panacea for engineering problems as a portfolio of paradigms”* [4].

Ultimately, design in the field of structural engineering is based on the same laws of physics as in nature, and because of this, similar problems, analogies, and models exist [1]. As pointed out by R. Aroca:

*“The basic structural constraints (gravity, wind and snow loads) are the same for natural structures as for buildings”* [5].

### 3.3. Transfer of Strategies from Nature to Structural Engineering

In natural models, there is an effective integration of form, function, and structure, as well as of these factors with their environment. This also represents one of the main objectives of structural design [6].

The transfer of models from nature to the field of engineering makes sense since they are the result of an extremely complex evolutionary process. This great complexity is on the other hand what makes their direct application, i.e., mere formal copying, to engineering problems very difficult.

In any case, this transfer of natural models to the field of structural engineering is an extraordinarily complex process in which the boundaries between the areas of knowledge of biology and engineering must be rigorously explored [7]. Interdisciplinary work is essential.

Despite the undeniable similarities mentioned above, there are also important differences: organisms in nature must be efficient in terms of energy to survive; they do not waste energy, and they need to operate with high effectiveness. Structural engineering does not necessarily have to be energy-efficient [1], although it is clearly a quality that is becoming increasingly important in the current context of climate change.

Design in nature is based on the unstoppable and endless gradual process of refinement and optimization. It takes an enormous amount of time, in the order of hundreds of thousands of years, often millions, to produce significant design changes [8]. The steps in this evolution are very small but constant and highly complex, incorporating an enormous amount of information, based after all on natural selection. There are no major milestones in the process but a huge accumulation of tiny advances [9]. 

Innovation in structural engineering is radically different. Breakthroughs are much more infrequent and come from ideas that involve major changes. In many cases, they do not arise from a clear precedent and have a much greater component of creativity and invention. Indeed, invention from scratch is possible [1].

Some authors argue for the superiority of natural design. Thus, in Luigi Colani’s words:

*“Whenever we talk about biodesign we should simply bear in mind just how amazingly superior a spider’s web is to any load-bearing structure man has made—and then derive from this insight that we should look to the superiority of nature for the solutions. If we want to tackle a new task in the studio, then it’s best to go outside first and look at what millennia-old answers there may already be to the problem”* [10].

Ricardo Aroca also highlights this idea of the supremacy of natural design:

*“The materials and structural forms of living things are difficult to match even by today’s technology; both in physical properties and design efficiency, their study can teach us many things we do not yet know”* [5].

Is it always beneficial to try to emulate nature in the search for optimal and efficient structures? We should not lose sight of the great risks involved in this process of mimesis, given its enormous complexity and the existence of important distorting factors such as scale, different materials, and different requirements for different structures.

In brief, the real challenge of biomimetics in the field of structural design is to try to apply the accumulated knowledge, tremendously optimized because of a long evolution, without falling into mere copying or direct translation of shapes [1]

### 3.4. Analogy as Biomimetic Model

Analogy can be considered the simplest strategy for the translation of ideas or forms from nature into architectural or structural design. It can be considered as a prescientific phase of research and should therefore be taken as a starting point and not as a point of arrival. Analogy is fundamentally based on looking for similarities, correlations, and equivalences in formal or functional terms. Since similar functions usually require similar formal approaches, analogy can be used as a germ for further in-depth research into many other aspects, as discussed above. Analogy between different fields of knowledge is conducive to innovation [1].

Frei Otto said about analogy:

*“Objects can be similar are equal in form, shape, construction, structure and material. They may have acquired this analogy through identical, similar or completely different development processes. The development processes play a key role in research of analogies. Typical technical and artificial products differ from creations in animate nature by a basically different development process. However, the process of selection often is very similar... crude, and artificially drawn analogies are called ‘trivial analogies’”* [11].

As will be discussed in the next section, analogy has been the main biomimetic strategy employed for the architectural and structural design of the Sydney Opera House.

### 3.5. Natural Shells vs. Concrete Shells

As mentioned, structures in nature are highly efficient systems due to their evolutionary character. In the case of shells, they are among the most efficient structural elements in nature because of their high resistance, minimum material, large spans, and sheltering characteristics. They are systems that can bridge a certain distance in a tremendously efficient manner, based on the adoption of the antifunicular shapes of the loads, which ensures the overall behavior of the structure under in-plane tensions only. In this way, it is possible to obtain extremely slender structures, which also means a minimum consumption of materials.

**Figure 2:**
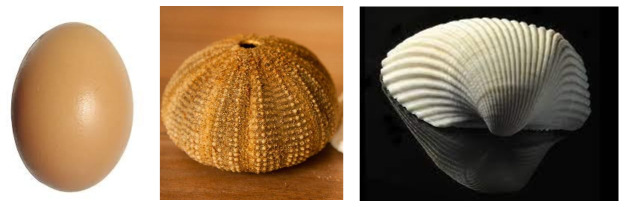
**Left**: eggshell (picture by www.pxhere.com; last accessed 24 November 2021). **Center**: seashell (picture by www.pixnio.com; last accessed 24 November 2021). **Right**: clam shell (photo by Bill Gracey; www.flickr.com; last accessed 24 November 2021).

There are many examples of shells in nature (Figure 2): eggshells, seashells, turtles, skulls, nuts, etc. [8]. Shells and exoskeletons protect the inner organs of sea urchins, snails, mussels, insects, and many other animals against predators and other potential dangers [12]. These natural forms have served as a source of inspiration for many architects and engineers throughout history. In particular, the unique characteristics of these constructions created by chickens, clams, scallops, and other mollusks can be considered as the origin of the construction of reinforced concrete shells. 

Avian eggshells are amazingly strong structures despite their apparent fragility and low resistance to point loads. However, their resistance to distributed compressive loads is remarkably high. Ostrich eggs have been tested in the laboratory with failure loads of over 5 kN [13]. As will be discussed in the following section, it has also been shown that the strength decreases with scale [13]. In any case, eggshells are proportionally much thinner than any man-made concrete shell and withstand comparatively much bigger loads [5].

Concrete roof shells, which originated in the 1920s, take these natural structures as a reference point, creating large architectural spaces of the highest structural efficiency through precise shapes that ensure the in-plane behavior of the concrete used. It was the invention of reinforced concrete, a formable, robust, economical material with high compressive performance, which led to the emergence of an architectural trend that used the shell construction system as a symbol of identity. 

**Figure 3:**
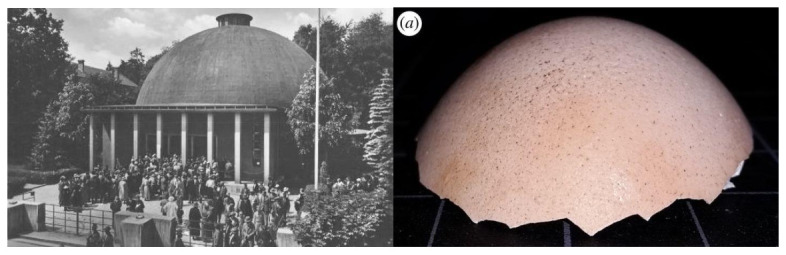
**Left**: Zeiss Planetarium in Jena, Germany, by Franz Dischinguer, 1926, (picture by Karl Müller, 1926). **Right**: intact egg cap resulting from distributed load experiment [12].

The first realizations of reinforced concrete shells were made by Franz Dischinguer and Walter Bauersfeld, based on the natural analogy with eggshells (Figure 3). Later, especially in the 1940s, 1950s, and 1960s, they became very popular with prestigious exponents such as Eduardo Torroja, Pier Luigi Nervi, and Felix Candela. Many of those designs were inspired by the structures in nature [14].

### 3.6. Influence of the Size

Size is an extremely influential factor, both in nature and in structural design. Therefore, the scale of things must be taken into consideration when trying to imitate natural designs [15]. 

The relationship between length, surface, and volume is not linear, and all physical processes are affected by this [8]. The relation between surface, volume, and specific weight implies that a system doubled in length is around eight times as heavy [8]. Large systems are much more strongly affected by the force of gravity, and therefore in these cases, the influence of the structure’s own weight is much more relevant. Thus, the smaller the size of the structure, the less relevant is the role of the force of gravity, and other forces such as surface tension take over the main role [8]. In many cases, phenomena from nature where molecular forces are most important cannot be scaled up [1].

Thompson explains this consideration very clearly in the following words:

*“Again, since the weight of a fruit increases as the cube of its linear dimensions, while the strength of the stalk increases as the square, it follows that the stalk must needs grow out of apparent due proportion to the fruit: or, alternatively, that tall trees should not bear large fruit on slender branches, and that melons and pumpkins must lie upon the ground”* [16].

In particular, curved structures such as shells have stresses proportional to the radius of curvature, and maintaining a constant thickness/radius of curvature ratio results in a proportional increase in weight, so a design is highly size-conditioned. Therefore, the transposition of shapes from nature to structural engineering in this field is complicated to say the least [5].

We could say that we cannot look for specific formal answers to imitate in nature; rather, it can be deduced in general terms that a multitude of solutions to the same problem are possible, and the intervals of validity in the structural field are closely linked to the sizes [5].

Thus, when in an attempt to mimic a natural design it is necessary to resize any phenomenon, it is essential to take into consideration the important effect of these differences. As will be explained below, this is a critical factor in the case of the Sydney Opera House’s structure.

## 4. Sydney Opera House: Inspiration by Nature

### 4.1. Introduction

The Sydney Opera House is one of the most iconic buildings of the 20th century, nominated as a UNESCO World Heritage Site in 2007. Its design process is also a very interesting case study in the use of references to nature, as will be shown below. 

When in 1956 Jørn Utzon developed his proposal for the International Architecture Competition for a new opera house in Sydney, he was very clear about both his working method and his references. As for the latter, he himself has stated in numerous interviews his predilection for natural references, with a strong preference for organic forms, thus distancing himself from the Modern Movement, the architectural style that clearly prevailed at the time.

The duality between the organicist (Art Nouveau, Organicism) and functionalist (Modern Movement) currents was evident in those days. The organicists tried to transfer elements directly from nature to be applied to buildings, opting for fluid geometries. On the other hand, the functionalists rejected the use of organic forms in favor of more Cartesian geometries and for giving total primacy to programmatic and functional aspects, perhaps to the detriment of other more “sensorial” ones.

In any case, in the 1950s there was a certain revival of organic architecture, encouraged by the strong development of reinforced concrete technology during the Second World War, with references such as Pier Luigi Nervi, Eduardo Torroja, and Félix Candela exploring new formal possibilities for the use of concrete.

Thus, the design proposed by Utzon for the Sydney Opera House competition can be clearly framed in this context. It is important to underline that this set of experimentations in the field of the formal possibilities of concrete are not produced as a mere “formalism” but are developed in the context of rigorous geometric studies (minimum energy surfaces) and form-finding (3D antifunicularity studies) to progressively refine the tools that ensure geometries that favor an optimal use of materials. In this context, mimesis with natural forms was also an important design tool.

### 4.2. Inspiration by Nature in the Competition Proposal

In 1956, after the recent Olympic Games for Melbourne, Australia was trying to maintain its momentum on the international scene by launching an international ideas competition for the construction of a large opera house in Sydney. Thus, on 29 January 1957, the competition panel ruled in favor of the entry presented by a very young and inexperienced Danish architect, who decades later would establish himself as one of the most relevant figures in his field, upon receiving the Pritzker Prize in 2003: Jørn Utzon.

Utzon developed his proposal mainly through physical models, trying to emphasize the three-dimensionality of his building, something that at that time could not be done in any other way, due to the lack of current digital design tools. He himself declared after winning the competition that he had worked as a sculptor, physically “shaping” his building. This idea of an object, a sculpture, a building in which the facades and roofs are diluted and intermingled, is also evident in his famous quote:

*“God sees from everywhere”* [17].

Utzon’s search for natural references for the development of his project was a constant: the shape of the roofs, the geometry of the facade, the chromatic range selected for the cladding, etc. Nature was his main source of inspiration, but he was neither focused on the forms produced by nature nor tied to organicist aesthetics but rather interested in the generating principles of nature [18]. As he later stated in several interviews:

*“I looked at flowers and insects, at organic forms. I wanted something that was growing out”* [19].

**Figure 4:**
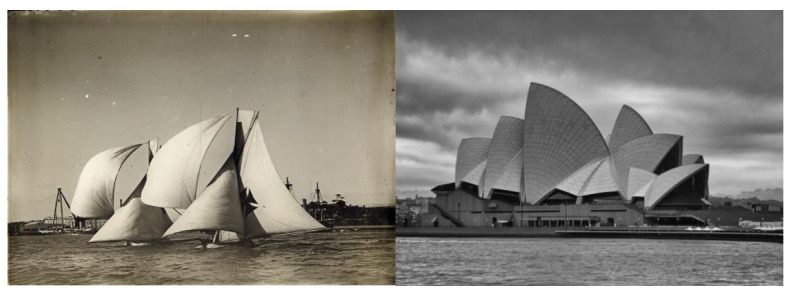
**Left**: boat with its sails unfurled (https://www.breizhskiff.com/tag/bethwaite/; last accessed 29 October 2021). **Right**: a hypothetical inspiration for Sydney Opera House (Photo by Douglas Banard; www.fineartamerica.com; last accessed 29 October 2021).

He himself acknowledged that it was the study of Sydney’s navigational maps that was his starting point and initial inspiration. In any case, regarding the possible source of inspiration for his formal proposal, in an interview published on 31 October 1992 in *Good Weekend* magazine [20], Utzon refutes the hackneyed explanation of the sails of the ships sailing in the bay (Figure 4):

*“Many people say my design was inspired by the sailing yachts in the harbour or by seashells. This is not the case. It is like an orange, you peel an orange, and you get these segments, these similar shapes. It was like this in my models. It was not that I thought it should be like sails in the harbour. It just so happened that the white sails were similar. I was influenced by the sails only to the extent that my father was a naval architect, and I was familiar with big shapes”* [19].

It should be noted that these explanations seem unlikely in any case, given that it seems to be proven that the orange as a source of inspiration for the resolution of the concrete shell roofs did not occur until 1962, 5 years after the competition proposal. In any case, Utzon also pointed out:

*“It is fine that people find what things are from what they see. Of course, they are like sails but this is not what we meant here, but I am very happy people think this”* [19].

As mentioned, Utzon began working on his proposal for the Sydney Opera House competition strongly influenced by the recent popularization of reinforced concrete shell roofs. It is therefore a design framed within what could be considered the “organicist alternative” to the prevailing mechanistic and rationalist current represented by the almost hegemonic Modern Movement within the architectural panorama of the time [1].

It is important to note that he worked during the competition phase without advice from structural engineering consultants. The competition proposal consists of 3-inch-thick reinforced concrete shells for the main roofs. As explained, is a very efficient typology in terms of material consumption and consequently allowed roofs to be built for large spaces at very low costs. The competition panel itself adduced as one of the main reasons for choosing Utzon’s proposal that it had been considered the most economical option among those analyzed.

**Figure 5:**
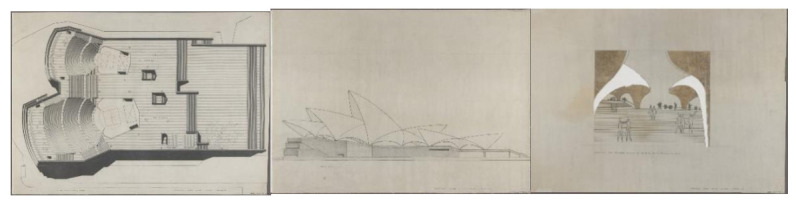
Original competition drawings for which the assessors of the Committee awarded Jørn Utzon first prize of GBP 5000 on 29 January 1957 (www.records.nsw.gov.au; last accessed 27 September 2021) [21].

Utzon therefore designed the curved surfaces that would form the roofs of the different parts of his building, imagining that they would be made of concrete, using shell structures a few centimeters thick, as eggshells. This is reflected in the competition drawings (Figure 5). However, as opposed to the aforementioned examples of Torroja, Candela, Nervi, etc., Utzon did not take geometry as the starting point for his creative process, but rather drew a series of organic forms, inspired by nature, that did not obey any geometrically known or mathematically defined figure. He himself referred to this fact in his competition report, stating that he had refused to use orthogonal forms in order to create what he called sculptures, large sculptures. These first natural references seem to be applied as formal analogies from a purely artistic point of view, without any great technical rigor to support them.

**Figure 6:**
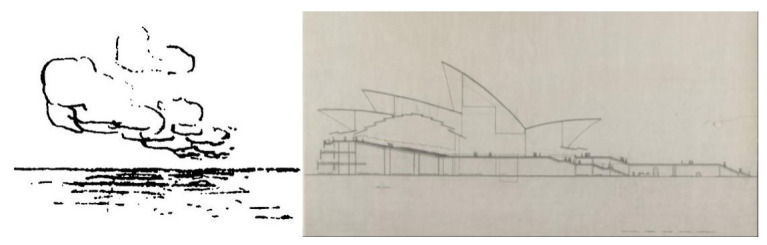
**Left**: Jørn Utzon sketch: platforms and plateaus (Jørn Utzon). **Right**: section from the competition drawings (Jørn Utzon; www.records.nsw.gov.au; last accessed 27 September 2021) [21].

The general profile of the roof pieces in the competition proposal is markedly horizontal, except for the larger pieces covering the auditorium areas, which are more markedly vertical. The surfaces of the roofs have smooth, rounded shapes, trying to provide an image of lightness despite the material with which they were conceived (reinforced concrete). For Utzon, again drawing on natural references, the roofs were like clouds of concrete (Figure 6), which should float above the landscape, contrasting with the heaviness of the base on which they rested. In Utzon’s words, the base was anchored to the earth and the roofs connected with the sky. Between these two zones, an area was established which in the drawings of the competition proposal appears apparently empty and diaphanous, in order to emphasize the tension between the two antagonistic concepts mentioned above. To this end, Utzon initially conceived all the building’s enclosures in glass, a large proportion of which were also mobile, thus maximizing the lightness of the hypothetical concrete shell. 

The only condition imposed by the design of a roof of this type is to be absolutely devoted to the antifunicular geometries that would ensure membrane behavior without the presence of significant bending moments. However, Utzon did not take geometry as the basis of his creative process, but “sculpted” through his physical models certain organic forms for the roofs. These shapes did not obey any geometrically known or mathematically defined form, and this aspect greatly conditioned the course that the development of the design was to take [19].

Ultimately, the great power of Utzon’s idea won the architectural competition, not without controversy: the press announced its surprise, and great figures of the international architectural scene such as Frank Lloyd Wright publicly expressed their rejection.

### 4.3. Inspiration by Nature during the Project Development

Aware of the construction challenge that lay ahead and of Utzon’s total inexperience, the committee appointed the engineer Ove Arup to take over the structural design. Arup was, at 64, a celebrity in the field of structural engineering. His Danish origin and his extensive experience in singular architecture projects led the Committee to hire his engineering firm.

The initial challenge for Arup and his team was not even trying to check through structural calculations whether Utzon’s proposal was feasible; the first challenge was “simply” to be able to translate these shapes into drawings. As indicated, the sketches that Utzon presented in the competition panels were based on the direct translation of the forms that he had arrived at through physical models. These drawings of plants, elevations, and sections were made freehand without any geometric rigor, so they actually wanted to express approximately certain shapes, but they were neither coincident with each other (plants, elevations, sections) nor reproducible through known mathematical expressions (circles, ellipses, parabolas, etc.) nor, therefore, ultimately, constructible [22].

This problem, difficult to understand nowadays due to the proliferation of all kinds of digital tools, was brilliantly expressed in 1983 by the architect Enric Miralles in his essay “How to Lay Out a Croissant” [23], in which he reflected on the enormous complexity of such a curious task with the tools available at the time. 

A few decades later, Frank Gehry solved an analogous situation in the case of the Guggenheim Museum in Bilbao with the help of 3D laser scanners and three-dimensional digital design software. With these new tools, he could directly transfer the geometry of his physical models to representable and buildable digital models, also using the so-called NURBS (non-uniform rational B-splines), curves that allowed the representation of free forms and that had been discovered a few years before, in the context of the automotive industry. However, in 1957 none of these digital tools existed, so the first thing that seemed clear was that the geometry of the roofs had to be transformed into some of the mathematically known forms at the time.

The great task in which everyone was involved at that time was to find a mathematical definition that reasonably resembled the natural shapes conceived by Utzon. The objective was clear: it was essential to find an analytically definable form that would also behave as closely as possible to that of a membrane, thus eliminating undesirable bending moments and making it possible to build a concrete shell with a reduced thickness. Utzon was obviously not aware of these constraints when he drew his proposal for the competition. He envisaged a beautiful sculpture, not a structurally efficient form.

**Figure 7:**
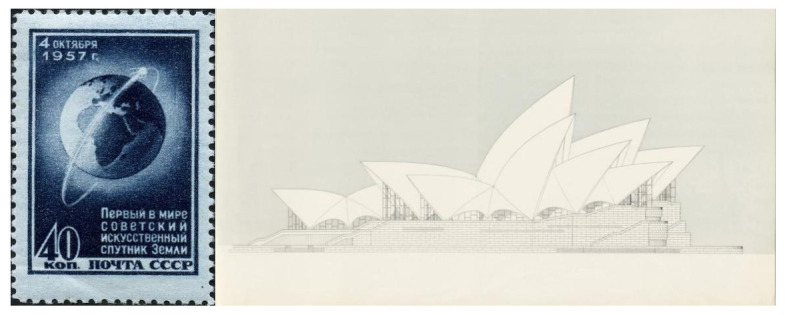
**Left**: image of the orbit traced by Sputnik space satellite, launched by the Soviet Union on 4 October 1957 (www.wikipedia.org; last accessed 4 December 2021). **Right**: Sydney Opera House’s roof profiles as defined on the Red Book, 1958 (www.records.nsw.gov.au; last accessed 4 December 2021) [21].

Throughout this process of searching for a feasible solution, natural references were also recurrent on Utzon’s side. Thus, Utzon, fascinated by images of the Soviet Union’s launch of Sputnik on 4 October 1957, immediately contacted Arup to express his interest in the silhouettes of the decks having the same geometry as the space rocket had described in its trajectory into space: sections of parabolas and ellipses, with a noticeably vertical outline at the start from the podium and gradually more horizontal as it ascended to its crown (Figure 7).

Although Utzon was unfamiliar with the geometrical fundamentals of correctly representing a parabola, he drew it freehand as best he could and sent it to Arup with a message indicating that this was the shape he wanted for the roofs. Based on the drawings in the 1958 “Red Book”, a new set of plans was drawn up, finalized in December 1960, in which both the ridge tiles and the cross-sections (hypothetical ribs) of the roofs were parabolic in shape. The drawings already showed a structural scheme consisting of two concrete sheets joined together by two families of beams in perpendicular directions. The two halves of each roof were tied together by concrete walls at the end facades. In this way, all the roof parts were interlocked with each other. As can be seen from the drawings presented, the total thickness of the concrete sheets was kept at approximately 15 cm (7.5 + 7.5 cm, including the ceramic pieces that would make up the exterior finish), the total thickness of the roof being 1.5 meters [24]. 

This typological solution was soon discarded due to its impossibility of being structurally analyzed, even with the help of the first computers available at the time. Circular and elliptical shapes were also tested, considering both metal and concrete structures, to form a graphic catalog of up to 12 solutions studied. In general, these are attempts to force the structure into a known shape.

**Figure 8:**
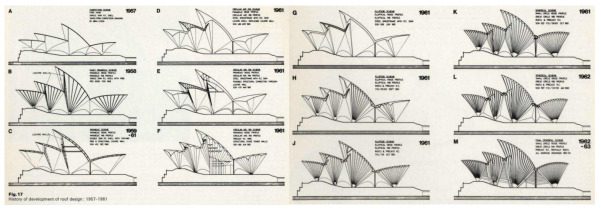
Temporal evolution of the different alternatives studied for the roof structure (*The Arup Journal*, 1973) [25].

Thus, over 5 long years, from 1957 to 1962, Arup and his team tried unsuccessfully to establish a valid geometry for the different volumes of the building [25]. Parabolic, elliptical, and similar geometries were studied (Figure 8). Systematically, the proposals were rejected by Utzon, who viewed with great suspicion that the sculptural forms inspired by nature to which he had arrived with his initial models would be modified. In the face of the despair of the Arup team, Utzon vehemently persevered with his idea, with lapidary phrases such as:

*“We can go to the Moon... of course we can build this building”* [19].

The situation was very tense. Ronald Jenkins, the project manager within the Arup team, a few months before he resigned from further involvement in the project in 1961, stated:

*“We went to all this trouble because of the shells being the wrong shape as we pointed out to you right at the beginning”* [19].

His major objection to Utzon’s original design was that the shapes of all the shell parts were different from each other. In addition to this, the fact that they had no defined geometry made it impossible to reuse the formwork, which in turn drove up construction costs.

A few months later Arup presented two alternatives to Utzon; the first consisted of “V”-shaped concrete ribs, taking up the idea used on the ground floor beams, consisting of folds in a continuous concrete surface, but with the added complexity of having a two-way curvature in the case of the roof beams. The use of steel was thus dispensed with, returning to a solution of reinforced concrete only.

Utzon received Arup’s proposal loud and clear:

*“I don’t care what its costs. I don’t care what scandal it causes; I don’t care how long it takes, that is what I want”* [19].

Utzon was not very much in favor of the use of a “hidden” steel structure inside the concrete structure as he considered it to be a “dishonest” gesture. On the contrary, the triangular concrete beams seen from the inside clearly showed the load path, which he considered essential for an acceptable solution for the roof structure. Thus, the family of concrete beams arranged in a fan-shaped pattern, concentrating at their confluence at the supports, was completely to his liking and immediately met with his approval.

### 4.4. Inspiration by Nature in the Final Solution: The Orange Analogy

Having established, at the typological level, the fan-shaped concrete rib scheme as the solution agreed as valid by all parties, the geometrical part of the problem remained to be solved: what shape should be given to the surfaces so that they would be geometrically representable, provide a correct structural performance, and also provide a simple construction?

Utzon did not have the technical background to solve a problem of this magnitude, yet it seems that he was the one who quite by accident came up with the solution to a problem that some of the leading engineers of the day had been grappling with for several years. It certainly seems clear that Utzon would have been unable to find the solution, even if this discovery had been largely fortuitous, without the “training” that the Arup engineers had unwittingly given him through the countless meetings they had held on the subject to try to make Utzon clearly understand the problem they were facing. It is well known that the correct formulation of a question is the best basis for finding an answer to it, and this part had certainly been to the credit of the Arup team.

At this time of maximum pressure, when the urgency to finally find a viable solution for the roof construction was pressing, the search for inspiration in nature remained the main driving force for Utzon.

Several years ago, Eero Saarinen, during a breakfast with Utzon, had explained the behavior of his concrete shell roofs for the TWA building by cutting a grapefruit and showing the shapes of its envelope.

**Figure 9:**
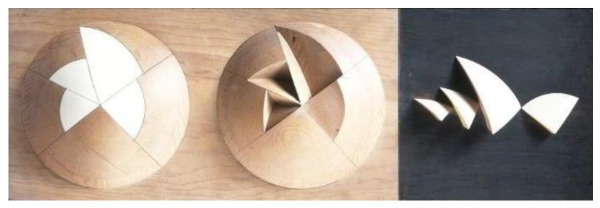
Wooden model with the deck pieces taken as surfaces of the same sphere (picture by MoMa, 1961; www.moma.org; last accessed 2 December 2021).

There is no unanimous version of these facts, but apparently, Utzon, very angry with the way things were going and frustrated that his life’s project was doomed to failure, went into the factory in Hellebaek where all the models of the project were displayed. He began to stack the shells of the large model to make space when he noticed how similar the shapes appeared to be. He noticed that they fit together perfectly, like a Russian doll. As a result of this fortuitous discovery and asking himself about the geometric shape that generates different curvatures from the same radius, he realized that this geometric figure was the sphere. So, each roof could perhaps be derived from a single, constant form, such as the plane of a sphere (Figure 9).

Moved by the great excitement that this discovery had generated in him, he immediately began to experiment with spheres. Given his limited knowledge of geometry, he began by working with his children’s plastic beach ball. He used water to draw different shapes on the surface of the ball, which, when dry, became visible through a noticeable change in color. In this way, he was able to experiment with the possible surfaces that could be generated from the same sphere. Once he was satisfied with his experiments with the beach ball, Utzon went to the shipyard in Helsingor, where he usually made his models, to build one with his new spherical shapes, and at the same time, he called an urgent meeting with Arup. 

Ove Arup summarizes in the following words, taken from a conference for the Prestressed Concrete Development Group read in London in 1965, the changes on the shell roofs proposed by Utzon:

*“Then Utzon called from Copenhagen saying that he had solved the whole previous problem. The point was to change the whole shape of the shells by the cut generated by the sphere itself. So now all the shells were spherical, and their ribs followed the meridian curves, on the sphere, of the same radius, 246 feet”* [26].

Arup immediately accepted the proposal and agreed to give the sphere a radius of approximately 74 meters (246 feet), which was the distance between the outer faces of the extreme ribs [27]. The adoption of a spherical shape allowed the use of a common formwork family for all parts, which would simplify and therefore greatly reduce the cost of roof construction. In addition, the calculation was also greatly simplified.

The explanation of how Utzon was able to come up with the idea of spherical geometry is, in his own words, as follows:

*“I’ve grown up in big shipyards and I had at Elsinore, close to my office, all the possibilities I wanted for studying the production of big, curved shapes.... Also, I had developed various systems for prefabrication in the building industry before the Opera House”* [28].

Given Utzon’s familiarity with prefabricated systems, he immediately proposed that the family of circular ribs be prefabricated in reinforced concrete, to which Arup, very sensitive to finding a simple and economical construction process, immediately agreed.

**Figure 10:**
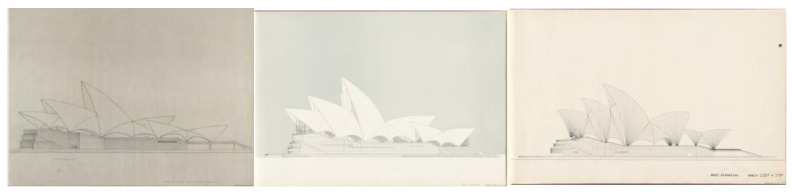
Design evolution. From left to right elevations drawing from 1956 (Brown Book, competition proposal), 1958 (Red Book, elliptical geometry), and 1962 (Yellow Book, spherical geometry) (www.records.nsw.gov.au; last accessed 2 December 2021) [21].

In January 1962, Utzon submitted his Yellow Book defining the new geometry of the roofs, details of the precast ribs, and the tiling. The proposal was very radical since, after long years of stubbornness, it represented a very strong geometric modification with respect to the competition proposal (Figure 10). On the other hand, it also included some positive aspects such as the relative ease of graphically representing the surfaces of the different volumes, as well as the possibility of prefabricating both the structure and the roof coverings, as there was already a single curvature. 

The final solution based on triangular-shaped folded concrete ribs was most likely influenced by the work of the Italian engineer Pier Luigi Nervi, and in particular by the building he constructed in 1960 for the Olympic Games in Rome: The Palazzetto dello Sport. This building covered a span of approximately 100 meters with folded, V-shaped reinforced concrete ribs only 9 cm thick.

Utzon himself reflected on this as follows:

*“Through my work with curved shapes in the opera house I have been inspired to go further into free architectural forms, but at the same time to control the geometry which makes it possible to erect the building out of mass-produced components. I am fully aware of the danger of using curved forms in contrast with the relative security of basing architecture on rectangular forms, but the curved form world offers something which one will never find in rectangular architecture. The ships’ hulls, the caves and the sculptures prove it”* [19].

This finding also allowed Utzon to move away from the expression of a style, in this case the concrete shell architecture, so fashionable at the time, and towards something more timelessly universal, based on purely geometric concepts inspired by natural forms.

Thus, the final roof structure was based on large precast concrete ribs, up to 3 m deep, joined together by post-tensioning and epoxy resins [29]. Obviously, the image of the roof structure had nothing to do with Utzon’s initial images, neither from a geometric point of view nor from a typological point of view. Both public opinion and the Australian press began to form a very critical current with the project, mainly due to the change in its forms. In this context, on February 28 of 1965, Utzon sent a letter of resignation to the Committee, and a few days later he left Australia, a country to which he would never return, not even to see his building finished. Finally, on October 20, 1973, more than 11 years behind schedule and with a budget deviation of more than 1,000%, Queen Elizabeth II of England inaugurated the building. 

Once the roof structure had been structurally resolved and was self-supporting, the facades were freed from bearing any kind of stress other than that due to wind loads. With the concrete rib solution, it was no longer necessary to use the facades as tie rods to avoid the deformation that would occur when considering the roofs as concrete slabs, and the feeling of transparency that Utzon had longed for could be achieved [28].

**Figure 11:**
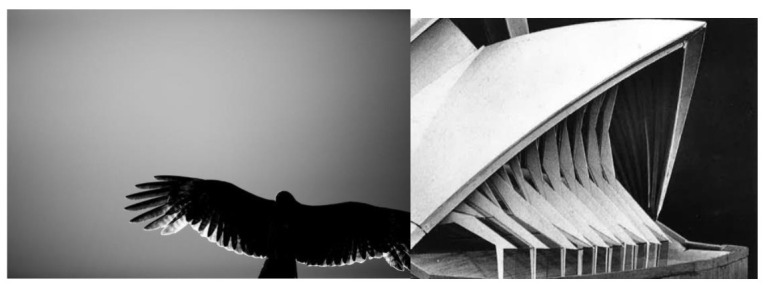
**Left**: image of a bird in flight, which Utzon used as a reference for the development of the facades (https://pxhere.com/es/photo/1224384; last accessed 2 December 2021). **Right**: glass wall design principle (Yellow Book, 1962); (www.records.nsw.gov.au; last accessed 2 December 2021) [21].

Once again Utzon turns to analogies with natural forms for his facade design. At first, Utzon proposed glass enclosures in vertical planes, but he soon realized that this type of enclosure would not produce the effect of transparency he was looking for, so he began to break up these vertical lines in search of more organic solutions, apparently inspired by the image of a bird in flight (Figure 11).

## 5. Results: Evaluation of Nature-Inspired Strategies in the Sydney Opera House Project

### 5.1. Feasibility Analysis of the Structure of the Competition Proposal

Despite nearly two decades of concerted efforts by some of the brightest architects and engineers, Utzon was finally unable to see the opera house he envisioned built: he conceived a building ahead of its time. The competition proposal had to be substantially altered. These alterations were mainly the consequence of a lack of sufficient scientific and technological means, as well as a poorly conditioned starting point from a structural perspective. The design, inspired by natural forms but based on pure translation by formal analogy, without taking into consideration both strong geometrical constraints and issues of scale, failed in its attempt to be built.

However, the question of whether Utzon’s original design for the roofs was feasible has so far remained unanswered, as it could not be tested at the time [30]. Based on the available competition documentation, represented by Utzon through two-dimensional projections of the building (floor and elevation drawings), a three-dimensional roof digital geometry has been reconstructed (using Rhinoceros), in particular of the main auditorium area. 

**Figure 12:**
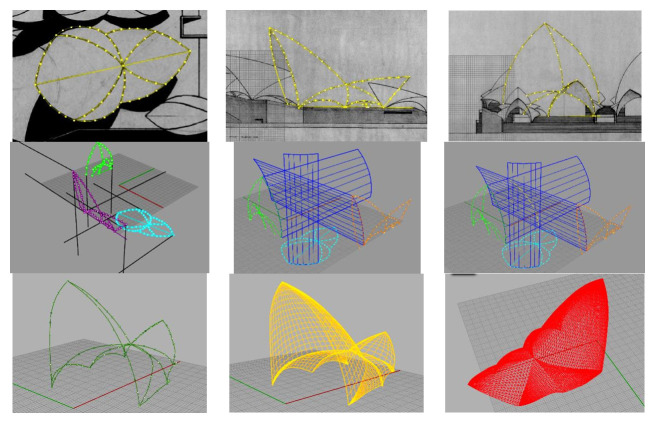
Geometry digitization process: point capture (**top**), edge identification (**center**), and surface and mesh generation (**bottom**).

The geometry generation procedure consisted of the following: First, competition plan and elevation drawings were digitized using Rhinoceros v5.0 software through a consecutive point capture (Figure 12, top). Curves were interpolated based on previously captured points (plan and elevation). Third-degree polynomials were considered for interpolation (Figure 12, mid-left). Surfaces were generated based on the obtained curves, projecting them in the main directions of the space (x, y, z) (Figure 12, mid-center). Edge curves were obtained from the intersection of the generated surfaces, constituting the master curves for the roof geometry (Figure 12, mid-right). From this set of space curves (Figure 12, bottom-left), surface fragments framed by said curves were generated (Figure 12, bottom-center). Once the surfaces were generated, a triangular mesh of three nodes triangular elements was applied for subsequent structural analysis (Figure 12, bottom-right). 

Geometry was imported into finite element analysis software Autodesk Robot Structural Analysis v12. Such software has been used to conduct the relevant structural analysis for defining the essential characteristics of the materials used, boundary conditions, applied loads [31], etc. 

The following load cases were considered: SW (self-weight; 12 cm thick concrete shell: 3.5 kN/m^2^), DL (dead load; tiles and mortar: 0.6 kN/m^2^), LL (live load; maintenance load: 0.4 kN/m^2^), Wx and Wy (wind along X- and Y-axes; Australian Standard CA34, Part II. SAA Loading Code, Part II: wind forces, dated 1971; C_p_ = 1.0 kN/m^2^, C_s_ = 0.5 kN/m^2^), T+20 (structure global temperature rise; +20 °C), and T-20 (structure global temperature decrease; −20 °C).

**Figure 13:**
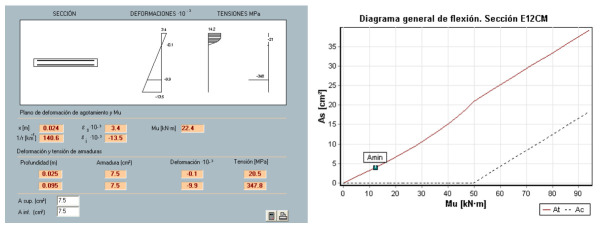

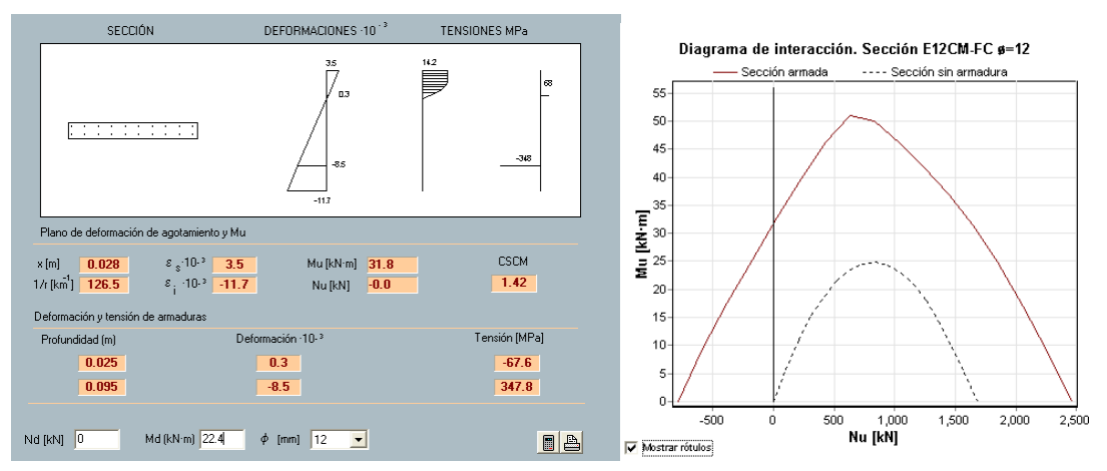
General bending diagram of a 12 cm thick reinforced concrete sheet. Prontuario Informático IECA (**top**). Interaction diagram axial-bending moment of a 12 cm thick reinforced concrete shell. Prontuario Informático IECA (**bottom**).

The bending moment capacity of the 12 cm thick concrete shell has been calculated approximately in order to compare it with the bending moments obtained from the calculation model developed. The estimated ultimate bending moment is M_u_ = 22.4 kNm (Figure 13, top). If we consider the concomitant action of this bending moment with an axial force, either tensile or compressive, we will be in a scenario much closer to the real one in the structure to be analyzed (Figure 13, bottom).

**Figure 14:**
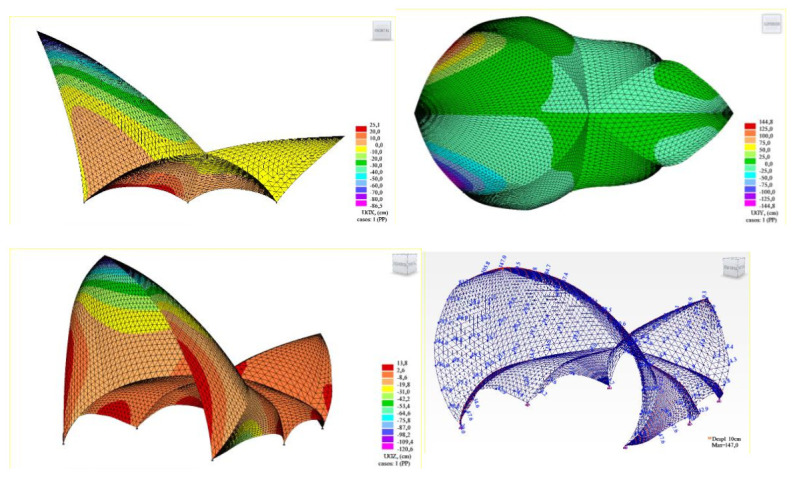
Node displacements under self-weight load along the global main axes X, Y, and Z.

An elastic linear analysis was conducted. The elastic properties of the concrete considered were as follows: E = 30 GPa, ν = 0.2, and density δ = 2500 kg/m^3^. Two-dimensional three-node triangular shell finite elements were used. Since self-weight is clearly governing and in order to have as clear results as possible, results are presented only for this load case (Figure 14).

Considering the obtained displacements (maximum instantaneous elastic displacements under self-weight along the global axes: dx = 86 cm; dy = 145 cm; dz = −120 cm), it can be concluded that Utzon’s competition geometrical proposal was not buildable due to inadequate structural performance. The roof’s structural behavior is clearly unacceptable, showing that the structure does not have sufficient stiffness and tends to “open” under the effect of its own weight.

**Figure 15:**
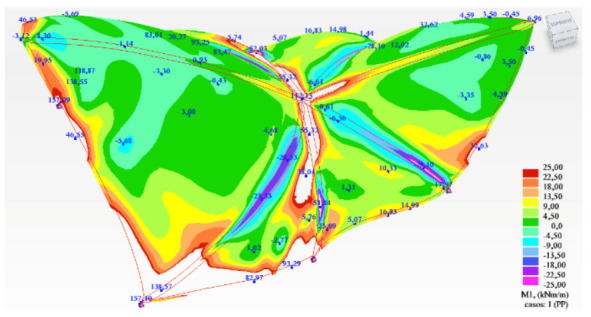
Bending moments according to the principal directions (M1) under self-weight load.

However, in order to enhance the understanding of the behavior of the structure under analysis, an image is provided showing the obtained bending moments according to the principal directions, M1 (Figure 15).

### 5.2. Study of the Effect of Scale on the Structure of the Competition Proposal

**Figure 16:**
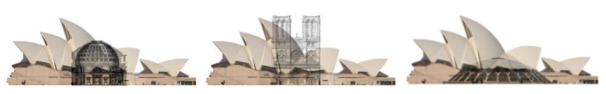
Sydney Opera House building size (67 m height above sea level, 186 m in length) compared to Pantheon (Rome, 27 B.C.; **left**), Notre Dame (Paris, 1345; **center**), and Palazzetto dello Sport (Rome, 1960; **right**).

In view of the above, it seems clear that the proposal Utzon submitted to the Sydney Opera House competition was not feasible as he envisaged it. The main problem, apart from the incorrect choice of building form, was the enormous size of the concrete shells, with parts up to approximately 45 meters high (Figure 16).

**Figure 17:**
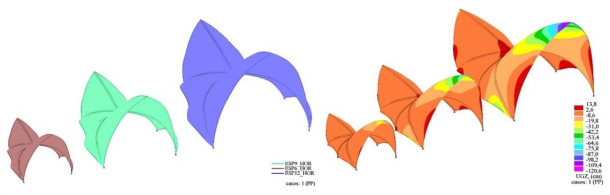
**Left**: full-scale calculation model (blue), scaled 75% (green) and scaled 50% (red). **Right**: displacements of the nodes of the structure in the global *Z*-axis for the self-weight load case.

In order to analyze the effect of the size of the building on the structural behavior of the decks, two additional models have been developed, consisting of 75% and 50% scaling, respectively, of the original calculation model presented above (Figure 17). The thicknesses of the concrete shell have been scaled accordingly, obtaining thicknesses of 9 and 6 cm, respectively.

**Figure 18:**
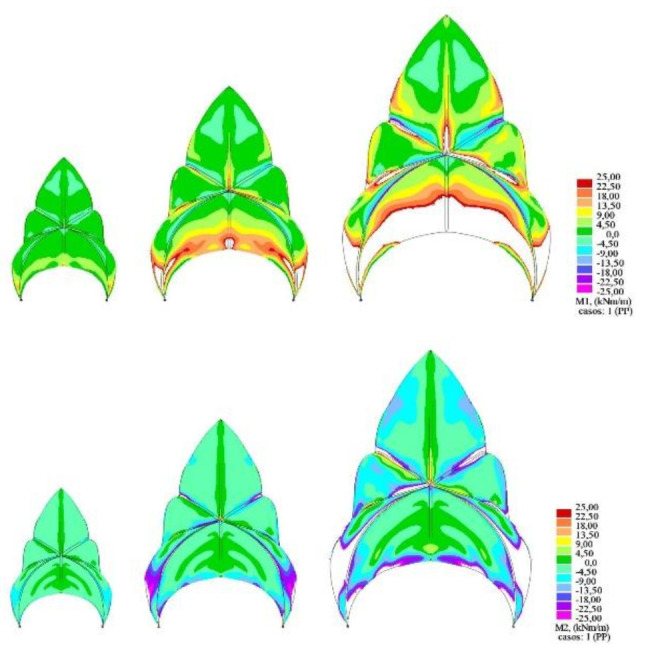
Principal bending moments M1 (**top**) and M2 (**bottom**). Load case: self-weight.

The impact that the change in the size of the structure has on both the maximum displacements obtained (Figure 17, right) and the bending stresses is evident (Figure 18).

In the above images, the color gradation has been scaled so that the values obtained are not drawn if they exceed the permissible simple bending value of approximately +/− 25 kNm. These blank areas are therefore areas of the roof structure that are not capable of resisting the acting internal bending moments. It can be seen that the overall performance of the structure is quite far away from a membrane behavior. 

The case of the Sydney Opera House shows us how the structural design is not initially contemplated but appears later, in a rather forced way, providing the necessary structural and constructive feasibility. In view of the eventful design and construction process of the building, this model can be considered inadequate, especially when it comes to the design and construction of a complex building. 

Felix Candela harshly summarized this question in 1968:

*“The example of the Sydney Opera House and, above all, the observation of the enormous difference between the slenderness and lightness of the original conception of the shells and the heaviness and complication of the final structure, should make us think about the convenience of counterbalancing the arrogant attitude towards architectural problems with a certain dose of humility and awareness of structural and human limitations”* [32].

## 6. Conclusions

Nature has always been an important attraction and source of inspiration in the field of architectural and structural design. Having at our disposal designs resulting from millions of years of evolution is an enormous opportunity. Unfortunately, this translation of form is by no means simple, and many factors, especially the scale, must be taken into consideration.

In particular, in the case of the structural design of the Sydney Opera House, inspiration from natural forms played a major role, both in the competition stage and during the almost 17 years of design and construction. However, this biomimicry was limited in most cases to simple formal analogies, which at first greatly conditioned the development of the project (even jeopardizing its viability) but which in the end paradoxically ended up also resolving the challenge. The orange analogy solved in a simple and effective way all the problems that Utzon and his team had been facing for 5 years.

Utzon’s stubbornness led to a long paralysis in the development of the project due to his initial erroneous choice of form for the roofs, based on a natural analogy. However, his genius resolved the complicated situation in time, paradoxically relying again on analogies with nature.

## Data Availability

The data used to support the findings of this study are included within the article.
